# Disentangling cardiovascular control mechanisms during head-down tilt via joint transfer entropy and self-entropy decompositions

**DOI:** 10.3389/fphys.2015.00301

**Published:** 2015-10-27

**Authors:** Alberto Porta, Luca Faes, Andrea Marchi, Vlasta Bari, Beatrice De Maria, Stefano Guzzetti, Riccardo Colombo, Ferdinando Raimondi

**Affiliations:** ^1^Department of Biomedical Sciences for Health, University of MilanMilan, Italy; ^2^Department of Cardiothoracic, Vascular Anesthesia and Intensive Care, IRCCS Policlinico San DonatoMilan, Italy; ^3^BIOtech, Department of Industrial Engineering, University of TrentoTrento, Italy; ^4^IRCS PAT-FBKTrento, Italy; ^5^Department of Electronics Information and Bioengineering, Politecnico di MilanoMilan, Italy; ^6^Department of Emergency and Intensive Care, San Gerardo HospitalMonza, Italy; ^7^IRCCS Fondazione Salvatore MaugeriMilan, Italy; ^8^Department of Emergency, L. Sacco HospitalMilan, Italy; ^9^Department of Anesthesia and Intensive Care, IRCCS Humanitas Clinical and Research CenterRozzano, Italy

**Keywords:** information dynamics, multivariate linear regression analysis, blood pressure variability, heart rate variability, baroreflex, cardiopulmonary coupling, autonomic nervous system

## Abstract

A full decomposition of the predictive entropy (PE) of the spontaneous variations of the heart period (HP) given systolic arterial pressure (SAP) and respiration (R) is proposed. The PE of HP is decomposed into the joint transfer entropy (JTE) from SAP and R to HP and self-entropy (SE) of HP. The SE is the sum of three terms quantifying the synergistic/redundant contributions of HP and SAP, when taken individually and jointly, to SE and one term conditioned on HP and SAP denoted as the conditional SE (CSE) of HP given SAP and R. The JTE from SAP and R to HP is the sum of two terms attributable to SAP or R plus an extra term describing the redundant/synergistic contribution to the JTE. All quantities were computed during cardiopulmonary loading induced by −25° head-down tilt (HDT) via a multivariate linear regression approach. We found that: (i) the PE of HP decreases during HDT; (ii) the decrease of PE is attributable to a lessening of SE of HP, while the JTE from SAP and R to HP remains constant; (iii) the SE of HP is dominant over the JTE from SAP and R to HP and the CSE of HP given SAP and R is prevailing over the SE of HP due to SAP and R both in supine position and during HDT; (iv) all terms of the decompositions of JTE from SAP and R to HP and SE of HP due to SAP and R were not affected by HDT; (v) the decrease of the SE of HP during HDT was attributed to the reduction of the CSE of HP given SAP and R; (vi) redundancy of SAP and R is prevailing over synergy in the information transferred into HP both in supine position and during HDT, while in the HP information storage synergy and redundancy are more balanced. The approach suggests that the larger complexity of the cardiac control during HDT is unrelated to the baroreflex control and cardiopulmonary reflexes and may be related to central commands and/or modifications of the dynamical properties of the sinus node.

## Introduction

Head-down tilt (HDT) is an experimental maneuver inducing an increase of the venous return, central blood volume, and central venous pressure (London et al., 1983; Nagaya et al., 1995). The resulting loading of the cardiopulmonary receptors in the right atrium and pulmonary veins leads to a sympatho-inhibitory response increasing the forearm blood flow (London et al., 1983; Nagaya et al., 1995; Tanaka et al., 1999) and decreasing the forearm vascular resistance (London et al., 1983; Nagaya et al., 1995; Tanaka et al., 1999), total peripheral resistance (Nagaya et al., 1995) and efferent muscle sympathetic nerve activity (Nagaya et al., 1995; Tanaka et al., 1999). Since the reduction of venous return and the consequent sinoaortic and carotid baroreceptor unloading during head-up tilt leads to a vagal withdrawal and sympathetic activation (Cooke et al., 1999; Marchi et al., 2013), to an engagement of the arterial baroreflex (Taylor and Eckberg, 1996; Porta et al., 2011) and to a reduction of the importance of the cardiopulmonary pathway (Porta et al., 2012b), it can be hypothesized that the acute central circulatory hypervoleamia induced by HDT produces the opposite cardiovascular response. Indeed, a reduced involvement of the cardiac baroreflex and an improved relevance of the cardiopulmonary circuits during HDT would explain the previously observed increase of respiratory sinus arrhythmia and the decrease of arterial pressure variability especially in the low frequency band (Porta et al., 2014b).

In the field of information dynamics multivariate tools have been recently devised that allow the quantitative description of the dynamical interactions among time series (McGill, 1954; Schreiber, 2000; Barnett et al., 2009; Faes et al., 2011, 2013, 2015; Lizier et al., 2011; Wibral et al., 2011, 2014; Chicharro and Ledberg, 2012; Stramaglia et al., 2012; Kugiumtzis, 2013; Porta et al., 2014a, 2015; Barrett, 2015). These tools have been successfully applied to disentangle physiological mechanisms from the spontaneous variability of the heart period (HP), systolic arterial pressure (SAP), and respiration (R). For example, the predictive entropy (PE) of HP assessed in the universe of knowledge Ω = {HP,SAP,R}, measuring the decline of uncertainty about the present HP due to the knowledge of the past history of HP, SAP, and R series, was taken as a measure of the loss of complexity of the cardiac neural regulation. Indeed, the PE of HP increased during the vagal withdrawal induced by an orthostatic challenge (Faes et al., 2015; Porta et al., 2015) and during an experimental maneuver imposing the regularization of the respiratory sinus arrhythmia (i.e., controlled respiration at slow breathing rate; Faes et al., 2015). The transfer entropies (TEs) from SAP to HP and from R to HP in Ω, denoting the portion of PE of HP solely attributable to past values of SAP and R respectively, were taken respectively as measures of the degree of involvement of the cardiac baroreflex and cardiopulmonary reflexes in controlling HP. Indeed, the TE from SAP to HP in Ω gradually augmented when the baroreflex was challenged in proportion to an orthostatic stimulus (Porta et al., 2015) and the TE from R to HP in Ω progressively decreased with age (Nemati et al., 2013; Porta et al., 2014a) as a likely consequence of the gradual vagal withdrawal inducing a progressive decoupling between HP variations and respiratory centers (Seals and Esler, 2000; Eckberg, 2003). Unfortunately, the full exploitation of this approach in assessing cardiovascular control is limited by the incomplete decomposition of PE of HP (McGill, 1954; Chicharro and Ledberg, 2012; Stramaglia et al., 2012; Barrett, 2015; Faes et al., 2015). Indeed, usually the full decomposition of PE is given for bivariate interactions (e.g., HP and R) (Faes et al., 2015) or limited to the TE term (Chicharro and Ledberg, 2012; Stramaglia et al., 2012; Barrett, 2015), while the full decomposition of the self-entropy (SE) has never been investigated.

The aim of this study is to provide a decomposition of PE including that of both TE and SE terms when the assigned target dynamic is HP and the two exogenous signals are SAP and R. The proposed decomposition is applied to elucidate the response of the cardiovascular control to HDT. We utilized: (i) the PE of HP as a global descriptor of the HP dynamic; (ii) the TEs from SAP to HP and from R to HP in Ω as markers of the degree of involvement of the cardiac baroreflex and cardiopulmonary reflexes, respectively; (iii) the SE of HP as a measure of the information stored into HP; (iv) the part of the SE of HP excluding the contributions of SAP and R as an index of the information stored into HP that cannot be explained by SAP and R; (v) the SAP-R interaction terms as measures of the redundant/synergistic contributions of SAP and R to the information transfer and storage. Results, over the same protocol exclusively relevant to the TEs from SAP to HP and from R to HP in Ω were presented to the EMBC 2014 (Porta et al., 2014b).

## Methods

### Modeling the linear variability interactions among HP, SAP, and R series

In the following we consider HP = *y* = {*y*(*n*), *n* = 1, …, *N*} as an effect series driven by a pair of exogenous series, *SAP* = *x*_1_ = {*x*_1_(*n*), *n* = 1,…,*N*}, and *R* = *x*_2_ = {*x*_2_(*n*), *n* = 1,…,*N*} where *N* is the series length and *n* is the progressive cardiac beat counter. The series *y, x*_1_, and *x*_2_ form the universe of knowledge Ω = {*y*,*x*_1_,*x*_2_} about the short-term control of the HP variability exploited in this study (Porta et al., 2012a). The samples of all series in Ω are normalized by subtracting the mean value and by dividing the result by the standard deviation, in such a way that *y, x*_1_, and *x*_2_ have zero mean and unit variance. We also define the following restricted universes of knowledge obtained from Ω by excluding both exogenous signals, i.e., Ω\*x*_1_,*x*_2_ = {*y*}, or one exogenous signals i.e., Ω\*x*_1_= {*y, x*_2_} and Ω\*x*_2_ = {*y, x*_1_}.

The open loop autoregressive (AR) model with two exogenous (X) inputs (ARX_1_X_2_) describes the dependence of the current value of *y, y*(*n*), on past values of the same signal and past values of the exogenous inputs *x*_1_ and *x*_2_ as

(1) $$\begin{array}{l} y\left(n\right)=\overset{p}{\underset{j=1}{\sum }}a_{j}\cdot y\left(n-j\right)\;+\;\overset{p}{\underset{j=\tau_{1}}{\sum }}b_{1j}\cdot x_{1}\left(n-j\right)\\ +\;\overset{p}{\underset{j=\tau_{2}}{\sum }}b_{2j}\cdot x_{2}\left(n-j\right)+w_{{\text{ARX}}_{1}{\text{X}}_{2}}\left(n\right) \end{array}$$

where *a*_*j*_, *b*_1*j*_, and *b*_2*j*_, with 1 ≤ *j* ≤ *p*, are the constant coefficients of the regression of *y* on past values of *y, x*_1_, and *x*_2_ respectively, *p* is the order of the regressions, τ_1_ and τ_2_ are the delays of the action from *x*_1_ and *x*_2_ to *y* respectively, and *w*_ARX_1_X_2__(*n*) is zero mean white Gaussian noise with variance $\lambda_{{\text{ARX}}_{1}{\text{X}}_{2}}^{2}$. In addition to the ARX_1_X_2_ model, four structures, all derived from the ARX_1_X_2_ model, are of interest in this study: (i) the ARX_1_ model in which the exogenous input *x*_2_ is disregarded by ignoring the regression of *y* on *x*_2_; (ii) the ARX_2_ model in which the exogenous input *x*_1_ is disregarded by ignoring the regression of *y* on *x*_1_; (iii) the X_1_X_2_ model in which the AR part is disregarded by ignoring the auto-regression of *y*; (iv) the AR model in which only the AR part is considered by ignoring both regressions of *y* on *x*_1_ and *x*_2_.

The one-step-ahead prediction of *y*, ŷ(*n*/*n*−1), based on the ARX_1_X_2_ model is given by

(2) $$\begin{array}{l} ŷ\left(n/n-1\right)=\overset{p}{\underset{j=1}{\sum }}{â}_{j}\cdot y\left(n-j\right)\;+\overset{p}{\underset{j=\tau_{1}}{\sum }}{\hat{b}}_{1j}\cdot x_{1}\left(n-j\right)\;+\overset{p}{\underset{j=\tau_{2}}{\sum }}{\hat{b}}_{2j}\cdot x_{2}\left(n-j\right) \end{array}$$

where the coefficients â_*j*_, ${\hat{b}}_{1j}$, and ${\hat{b}}_{2j}$, with 1 ≤ *j* ≤ *p*, are estimated according to an optimization criterion (here the least squares approach minimizing the variance of *w*_ARX_1_X_2__) (Soderstrom and Stoica, 1988). According to the same optimization criterion the one-step-ahead prediction of the considered simplified versions of the ARX_1_X_2_ model (i.e., the ARX_1_, ARX_2_, X_1_X_2_, and AR structures) can be analogously obtained after a new identification of the regression coefficients. The prediction error is defined as the difference between *y*(*n*) and ŷ(*n*/*n*−1). The ability of the model to describe the dynamic of *y* is quantified by the variance of the prediction error. It is bounded between 0 and the variance of *y*, σ^2^. Given the normalization of the series, the variance of the prediction error actually ranges between 0 and 1, where 0 indicates perfect prediction (the entire σ^2^ is explained by the model), and 1 indicates null prediction (no fraction of σ^2^ is explained by the model). In the following we will indicate the variances of the prediction error of the ARX_1_X_2_, ARX_1_, ARX_2_, X_1_X_2_, and AR models as $\sigma_{{\text{ARX}}_{1}{\text{X}}_{2}}^{2}$, $\sigma_{{\text{ARX}}_{1}}^{2}$, $\sigma_{{\text{ARX}}_{2}}^{2}$, $\sigma_{{\text{X}}_{1}{\text{X}}_{2}}^{2}$, and $\sigma_{\text{AR}}^{2}$ respectively.

### Definition of the information-theoretic quantities

**Figure 1:**
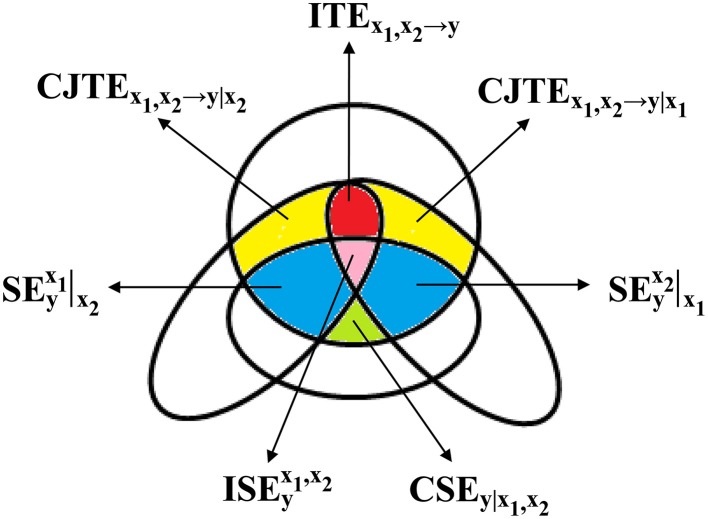
**A mnemonic Venn diagram, redrawn from Porta et al. (2015), of the information-theoretic quantities contributing to the decomposition of *PE*_*y*_ in Ω = {*y, x*_1_, *x*_2_}**. The diagram is devised to represent in the information domain the terms whose sum gives the *JTE*_*x*_1_, *x*_2_→*y*_ according to Equation (21), i.e., *CJTE*_*x*_1_, *x*_2_→*y*|*x*_1__ (yellow area), *CJTE*_*x*_1_, *x*_2_→*y*|*x*_2__ (yellow area), and ITE_*x*_1_, *x*_2_→*y*_ (red area), and the terms whose sum gives the SE_*y*_ according to Equation (22), i.e., CSE_*y*|_*x*__1_, *x*_2__ (green area), ${\text{SE}}_{y}^{x_{1}}{|}_{x_{2}}$ (blue area), ${\text{SE}}_{y}^{x_{2}}{|}_{x_{1}}$ (blue area), and ${\text{ISE}}_{y}^{x_{1},x_{2}}$ (pink area).

Under the hypothesis of linearity and Gaussianity of the dynamics, the Shannon entropy (ShE) of the normalized series *y* is ShE_*y*_ = 0.5 · log(2πe) (McEliece, 2002), where log is the natural logarithm, and the conditional entropy (CE) of y in Ω is ${\text{CE}}_{y}=0.5\cdot \text{log}\left(2\pi {\text{eσ}}_{{\text{ARX}}_{1}{\text{X}}_{2}}^{2}\right)$. ShE_*y*_ and CE_*y*_ measure, respectively, the total amount of information carried by *y* and its remaining portion that cannot be resolved using past samples of all signals present in Ω (Barnett et al., 2009). We define the PE of *y* in Ω (Chicharro and Ledberg, 2012; Faes et al., 2015) as

(3) $$\begin{array}{l} {\text{PE}}_{y}=\frac{1}{2}\text{log}\frac{1}{\sigma_{{\text{ARX}}_{1}{\text{X}}_{2}}^{2}} \end{array}$$

quantifying the portion of uncertainty of *y* that has been resolved in Ω (i.e., PE_*y*_ = ShE_*y*_−CE_*y*_); the SE of *y* in Ω\*x*_1_,*x*_2_ (Lizier et al., 2012; Wibral et al., 2014) as

(4) $$\begin{array}{l} {\text{SE}}_{y}=\frac{1}{2}\text{log}\frac{1}{\sigma_{\text{AR}}^{2}} \end{array}$$

measuring the part of uncertainty of *y* that has been resolved in Ω\*x*_1_,*x*_2_, (i.e., solely using past values of *y*); the conditional SE (CSE) of *y* given *x*_1_ and *x*_2_ (Porta et al., 2015) as

(5) $$\begin{array}{l} {\text{CSE}}_{y|x_{1},x_{2}}=\frac{1}{2}\text{log}\frac{\sigma_{{\text{X}}_{1}{\text{X}}_{2}}^{2}}{\sigma_{{\text{ARX}}_{1}{\text{X}}_{2}}^{2}} \end{array}$$

measuring the part of information carried by *y* that can be explained in Ω, above and beyond the one that can be resolved using past values of *x*_1_ and *x*_2_ (Figure 1); the conditional self-entropy (CSE) of *y* given *x*_1_ in Ω\*x*_2_ (Faes et al., 2015) as

(6) $$\begin{array}{l} {\text{CSE}}_{y|x_{1}}=\frac{1}{2}\text{log}\frac{\sigma_{{\text{X}}_{1}}^{2}}{\sigma_{{\text{ARX}}_{1}}^{2}} \end{array}$$

measuring the part of uncertainty of *y* that has been resolved in Ω\*x*_2_ (i.e., without accounting for the possible influences of the exogenous signal *x*_2_) above and beyond the one that can be resolved using past values of *x*_1_; the cross-entropy (C) from *x*_1_ to *y* in Ω\*x*_2_ (Faes et al., 2015) as

(7) $$\begin{array}{l} {\text{C}}_{x_{1}\to y}=\frac{1}{2}\text{log}\frac{1}{\sigma_{{\text{X}}_{1}}^{2}} \end{array}$$

measuring the fraction of information carried by *y* that can be explained in Ω\*x*_2_ given past values of *x*_1_; the joint TE (JTE) from *x*_1_ and *x*_2_ to *y* (Porta et al., 2015) as

(8) $$\begin{array}{l} {\text{JTE}}_{x_{1},x_{2}\to y}=\frac{1}{2}\text{log}\frac{\sigma_{\text{AR}}^{2}}{\sigma_{{\text{ARX}}_{1}{\text{X}}_{2}}^{2}} \end{array}$$

measuring the fraction of uncertainty of *y* that has been resolved in Ω above and beyond the one that can be resolved in Ω\*x*_1_,*x*_2_; the TE from *x*_1_ to *y* in Ω (i.e., by accounting for the possible influences of *x*_2_ on *y*) (Schreiber, 2000; Lizier and Prokopenko, 2010; Kugiumtzis, 2013), denoted here as the conditional JTE (CJTE) from *x*_1_ and *x*_2_ to y given *x*_2_ (Porta et al., 2015), as

(9) $$\begin{array}{l} {\text{CJTE}}_{x_{1},x_{2}\to y|x_{2}}=\frac{1}{2}\text{log}\frac{\sigma_{{\text{ARX}}_{2}}^{2}}{\sigma_{{\text{ARX}}_{1}{\text{X}}_{2}}^{2}} \end{array}$$

measuring the reduction of uncertainty of *y* that can be achieved when Ω\*x*_1_ is completed with the introduction of *x*_1_ (Figure 1); the TE from *x*_1_ to *y* in Ω\*x*_2_ (i.e., without accounting for the possible influences of *x*_2_ on *y*) (Faes et al., 2015) as

(10) $$\begin{array}{l} {\text{TE}}_{x_{1}\to y}=\frac{1}{2}\text{log}\frac{\sigma_{\text{AR}}^{2}}{\sigma_{{\text{ARX}}_{1}}^{2}} \end{array}$$

measuring the reduction of uncertainty of *y* that can be achieved when Ω\*x*_1_,*x*_2_ is enlarged with the introduction of *x*_1_. By reversing the role between *x*_1_ and *x*_2_, CSE_*y*|_*x*__2__, C_*x*_2_→*y*_, CJTE_*x*_1_, *x*_2_→*y*|*x*_1__ (Figure 1), and TE_*x*_2_→*y*_ can be computed as well.

### Redundant/synergistic contribution of x_1_ and x_2_ to JTE

The interactive TE (ITE) of *y* is defined as the variation between the sum of the information individually transferred from *x*_1_ to *y* and from *x*_2_ to *y* and the quote jointly transferred (McGill, 1954; Stramaglia et al., 2012) (Figure 1). Therefore, the following equality

(11) $$\begin{array}{l} {\text{JTE}}_{x_{1},x_{2}\to y}={\text{TE}}_{x_{1}\to y}+{\text{TE}}_{x_{2}\to y}-{\text{ITE}}_{x_{1},x_{2}\to y} \end{array}$$

provides a relation among the information jointly transferred from *x*_1_ and *x*_2_ to *y*, JTE_*x*_1_, *x*_2_→*y*_, the quantities individually transferred from *x*_1_ to *y*, TE_*x*_1_→*y*_, and from *x*_2_ to *y*, TE_*x*_2_→*y*_ and the ITE_*x*_1_, *x*_2_→*y*_. ITE_*x*_1_, *x*_2_→*y*_ < 0 implies synergy of *x*_1_ and *x*_2_ in contributing to JTE_*x*_1_, *x*_2_→*y*_, indicating that the information jointly transferred from *x*_1_ and *x*_2_ to *y* is larger than the sum of the information transferred from *x*_1_ to *y* and from *x*_2_ to *y* when *x*_1_ and *x*_2_ are taken individually. Therefore, the ITE_*x*_1_, *x*_2_→*y*_ measures the redundant or synergistic contribution of *x*_1_ and *x*_2_ to JTE_*x*_1_, *x*_2_→*y*_. The smaller and negative the ITE_*x*_1_, *x*_2_→*y*_, the more relevant the synergy of *x*_1_ and *x*_2_ in reducing the uncertainty about the present of *y* above and beyond the contribution of past values of *y*. ITE_*x*_1_, *x*_2_→*y*_>0 implies redundancy of *x*_1_ and *x*_2_ in contributing to JTE_*x*_1_, *x*_2_→*y*_, indicating that the information jointly transferred from *x*_1_ and *x*_2_ to *y* is smaller than the sum of the information transferred from *x*_1_ to *y* and from *x*_2_ to *y* when *x*_1_ and *x*_2_ are taken individually. Therefore, the larger and positive the ITE_*x*_1_, *x*_2_→*y*_, the more relevant the redundancy of *x*_1_ and *x*_2_ in reducing the uncertainty about the present of *y* above and beyond the contribution of past values of *y*. The ITE_*x*_1_, *x*_2_→*y*_ can be easily estimated as

(12) $$\begin{array}{r} {\text{ITE}}_{x_{1},x_{2}\to y}={\text{TE}}_{x_{1}\to y}-{\text{CJTE}}_{x_{1}x_{2}\to y|x_{2}}. \end{array}$$

Equation (12) clearly indicates that the information transferred to *y* in Ω\*x*_2_ might be larger or smaller than that in Ω depending on whether redundancy or synergy occurs. From Equation (12) it is clear that the sign of ITE_*x*_1_, *x*_2_→*y*_ depends on the balance between two quantities that are larger than 1: (i) $\sigma_{\text{AR}}^{2}/\sigma_{{\text{ARX}}_{1}}^{2}$; (ii) $\sigma_{{\text{ARX}}_{2}}^{2}/\sigma_{{\text{ARX}}_{1}{\text{X}}_{2}}^{2}$. If $\sigma_{\text{AR}}^{2}/\sigma_{{\text{ARX}}_{1}}^{2}$>$\sigma_{{\text{ARX}}_{2}}^{2}/\sigma_{{\text{ARX}}_{1}{\text{X}}_{2}}^{2}$, redundancy is present. Conversely, if $\sigma_{\text{AR}}^{2}/\sigma_{{\text{ARX}}_{1}}^{2}$<$\sigma_{{\text{ARX}}_{2}}^{2}/\sigma_{{\text{ARX}}_{1}{\text{X}}_{2}}^{2}$, synergy is detected. Since ITE_*x*_1_, *x*_2_→*y*_ is symmetric in *x*_1_ and *x*_2_, the abovementioned considerations hold by reversing the role of *x*_1_ and *x*_2._ The ITE_*x*_1_, _*x*__2_→*y*_ can be also expressed as a percent value with respect to JTE_*x*_1_, _*x*__2_→*y*_. This quantity will be denoted as ITE%_*x*_1_, _*x*__2_→*y*_.

### Redundant/synergistic contributions of x_1_ and x_2_ to SE

The SE_*y*_ can be seen as

(13) $$\begin{array}{l} {\text{SE}}_{y}\;=\;{\text{CSE}}_{y|x_{1},x_{2}}\;+\;{\text{SE}}_{y}^{x_{1},x_{2}} \end{array}$$

where

(14) $$\begin{array}{l} {\text{SE}}_{y}^{x_{1},x_{2}}\;=\;\frac{1}{2}\text{log}\frac{\sigma_{{\text{ARX}}_{1}{\text{X}}_{2}}^{2}}{\sigma_{\text{AR}}^{2}\cdot \sigma_{{\text{X}}_{1}{\text{X}}_{2}}^{2}} \end{array}$$

represents the synergistic/redundant contribution of *x*_1_ and *x*_2_ to SE_*y*_ (Figure 1). If ${\text{SE}}_{y}^{x_{1},x_{2}}$>0, then SE_*y*_ > CSE_*y*|_*x*__1_, _*x*__2__. This indicates that past values of *y* and of the exogenous sources, when taken together, contribute redundantly to resolve the uncertainty of *y* because the joint consideration of past values of *y* and of the exogenous sources worsens predictability of *y* compared to their separated observations. Conversely, if ${\text{SE}}_{y}^{x_{1},x_{2}}$ < 0, the joint knowledge of past values of y and of the exogenous sources improves prediction compared to their separated consideration, thus indicating that past values of *y* and of the exogenous sources contribute synergistically to reduce the uncertainty of *y*. The ${\text{SE}}_{y}^{x_{1},x_{2}}$ can be also expressed as a percent value with respect to SE_*y*_. This quantity will be denoted as ${\text{SE\%}}_{y}^{x_{1},x_{2}}$.

Defined the redundant/synergistic contributions of *x*_1_ and *x*_2_ to SE_*y*_ when *x*_1_ and *x*_2_ are individually considered as

(15) $$\begin{array}{l} {\text{SE}}_{y}^{x_{1}}\;=\;{\text{C}}_{x_{1}\to y}-\;{\text{TE}}_{x_{1}\to y} \end{array}$$

and

(16) $$\begin{array}{l} {\text{SE}}_{y}^{x_{2}}\;=\;{\text{C}}_{x_{2}\to y}-{\text{TE}}_{x_{2}\to y} \end{array}$$

the equality

(17) $$\begin{array}{l} {\text{SE}}_{y}^{x_{1},x_{2}}={\text{SE}}_{y}^{x_{1}}+{\text{SE}}_{y}^{x_{2}}-{\text{ISE}}_{y}^{x_{1},x_{2}} \end{array}$$

formally corresponds to Equation (11) as far as the information storage in *y* is concerned, where ISE stands for the interactive SE and represents the redundant/synergetic contributions of *x*_1_ and *x*_2_ to the information stored in *y*. At difference from Equation (11), where the ITE_*x*__1_, _*x*_2_→*y*_ is the unique synergistic/redundant term, in Equation (17) all parts might be positive or negative depending on whether *x*_1_ and *x*_2_, taken individually or jointly, contribute redundantly or synergistically to the information storage of *y*.

By substituting Equations (13), (15), and (16) into Equation (17) it can be easily demonstrated that the

(18) $${\text{ISE}}_{y}^{{\text{x}}_{1},x_{2}}{}_{y}=\frac{1}{2}\text{log}\left(\frac{\sigma_{{\text{ARX}}_{1}}^{2}}{\sigma_{{\text{X}}_{1}}^{2}\cdot \sigma_{\text{AR}}^{2}}\cdot \frac{\sigma_{{\text{ARX}}_{2}}^{2}}{\sigma_{{\text{X}}_{2}}^{2}\cdot \sigma_{\text{AR}}^{2}}\cdot \frac{\sigma_{\text{AR}}^{2}\cdot \sigma_{{\text{X}}_{1}{\text{X}}_{2}}^{2}}{\sigma_{{\text{ARX}}_{1}{\text{X}}_{2}}^{2}}\right)$$

thus leading to the following equality

(19) $$\begin{array}{l} {\text{ISE}}_{y}^{x_{1},x_{2}}={\text{C}}_{x_{1}\to y}-{\text{TE}}_{x_{1}\to y}-\left({\text{CSE}}_{y|x_{2}}-{\text{CSE}}_{y|x_{1},x_{2}}\right) \end{array}$$

that provides a viable estimate of ${\text{ISE}}_{y}^{x_{1},x_{2}}$.

### Full decomposition of PE of y in Ω

By following the definitions given in the previous sections it can be easily verified that the PE_*y*_ in Ω can be fully decomposed (Figure 1) as

(20) $$\begin{array}{l} {\text{PE}}_{y}={\text{JTE}}_{x_{1},x_{2}\to y}+{\text{SE}}_{y} \end{array}$$

(21) $$\begin{array}{l} {\text{JTE}}_{x_{1},x_{2}\to y}={\text{CJTE}}_{x_{1},x_{2}\to y|x_{2}}+{\text{CJTE}}_{x_{1},x_{2}\to y|x_{1}}+{\text{ITE}}_{x_{1},x_{2}\to y} \end{array}$$

(22) $$\begin{array}{l} {\text{SE}}_{\text{y}}={\text{CSE}}_{y|x_{1},x_{2}}+{\text{SE}}_{y}^{x_{1}}{|}_{x_{2}}+{\text{SE}}_{y}^{x_{2}}{|}_{x_{1}}+{\text{ISE}}_{y}^{x_{1},x_{2}} \end{array}$$

with

(23) $$\begin{array}{l} {\text{SE}}_{y}^{x_{1}}{|}_{x_{2}}+{\text{SE}}_{y}^{x_{2}}{|}_{x_{1}}+{\text{ISE}}_{y}^{x_{1},x_{2}}={\text{SE}}_{y}^{x_{1},x_{2}} \end{array}$$

(24) $$\begin{array}{l} {\text{SE}}_{y}^{x_{1}}{|}_{x_{2}}={\text{CSE}}_{y|x_{2}}-{\text{CSE}}_{y|x_{1},x_{2}} \end{array}$$

and

(25) $$\begin{array}{l} {\text{SE}}_{y}^{x_{2}}{|}_{x_{1}}={\text{CSE}}_{y|x_{1}}-{\text{CSE}}_{y|x_{1},x_{2}} \end{array}$$

where ${\text{SE}}_{y}^{x_{1}}{|}_{x_{2}}$ and ${\text{SE}}_{y}^{x_{2}}{|}_{x_{1}}$ represent the synergistic/redundant contribution of *x*_1_ conditioned on *x*_2_ and vice versa to SE_*y*_ in Ω (Figure 1). It is remarkable that the synergistic/redundant contribution of *x*_1_ and *x*_2_ to SE_*y*_, measured by ${\text{SE}}_{y}^{x_{1},x_{2}}$, depends on the balance among the synergistic/redundant contributions of one of the exogenous inputs conditioned on the other, i.e., ${\text{SE}}_{y}^{x_{1}}{|}_{x_{2}}$ and ${\text{SE}}_{y}^{x_{2}}{|}_{x_{1}}$, and on the synergistic/redundant contribution of *x*_1_ and *x*_2_ when they are jointly considered (i.e., ${\text{ISE}}_{y}^{x_{1},x_{2}}$). Even though Equation (23) formally corresponds to Equation (21) as far as the information storage in *y* is concerned, Equations (21) and (23) are structurally very different. Indeed, while all the terms in Equation (23) can be positive or negative, CJTE_*x*_1_, _*x*__2_→*y*|*x*_2__ and CJTE_*x*_1_, _*x*__2_→*y*|*x*_1__ in Equation (21) are positive (or null).

## Experimental protocol and data analysis

### Experimental protocol

We studied 13 healthy men aged from 41 to 71 years (median: 59 years). A detailed medical history and examination excluded the evidence of any disease. The subjects did not take any medication nor did they consume any caffeine or alcohol containing beverages in the 24 h before the recording. The study adhered to the principles of the Declaration of Helsinki for medical research involving human subjects. The human research and ethical review board of the “L. Sacco” Hospital approved the protocol. All subjects gave their written informed consent. Electrocardiogram (ECG) and noninvasive finger blood pressure (Nexfin, BMEYE, Amsterdam, The Netherlands) were recorded during the experiments. Signals were sampled at 400 Hz. Each experiment consisted of 10 min of baseline recording at rest in supine position (REST) followed by 10 min of recording during HDT with a table inclination of −25°. Before REST we allowed 15 min of stabilization. The recordings of the HDT session started 5 min after tilting the table. During the protocol, the subjects breathed according to a metronome at 16 breaths^.^min^−1^ to prevent modifications of the magnitude of the respiratory sinus arrhythmia owing to the changes of the breathing rate as much as possible (Hirsch and Bishop, 1981; Brown et al., 1993). All experiments were carried out in the afternoon in the same temperature-controlled room and the subjects were not allowed to talk during the protocol. Original data are available through the corresponding author.

### Extraction of the beat-to-beat variability series

After detecting the R-wave on the ECG and locating the R-wave peak using parabolic interpolation, the temporal distance between two consecutive R-wave apexes was computed and utilized as an approximation of HP. The maximum of arterial pressure inside the *n*-th HP [i.e., HP(*n*)] was taken as the *n*-th SAP [i.e., SAP(*n*)]. R signal was obtained from the respiratory-related amplitude modulation of the ECG. The amplitude of the first QRS complex delimiting HP(*n*), taken as the difference between the R-wave apex and the isoelectric line, was taken as the *n*-th R [i.e., R(*n*)]. The occurrences of R-wave and SAP peaks were carefully checked to avoid erroneous detections or missed beats. If isolated ectopic beats affected HP and SAP values, these measures were linearly interpolated using the closest values unaffected by ectopic beats. HP, SAP, and R sequences of 256 consecutive synchronous measures were chosen inside the REST and HDT periods, thus focusing on short-term cardiovascular regulatory mechanisms (Task Force of the European Society of Cardiology and the North American Society of Pacing and Electrophysiology, 1996). The random selection of the onset of analysis within the overall REST and HDT periods made this preprocessing step operator-independent. The series were linearly detrended before multivariate linear regression analysis. If evident nonstationarities, such as very slow drifting of the mean or sudden changes of the variance, were visible despite the linear detrending, the random selection was carried out again. The HP and SAP means and the HP and SAP variances were indicated as μ_HP_, μ_SAP_, $\sigma_{\text{HP}}^{2}$ and $\sigma_{\text{SAP}}^{2}$ and expressed in ms, mmHg, ms^2^, and mmHg^2^ respectively.

### Power spectral analysis

The power spectrum was estimated according to an univariate parametric approach fitting the series according to the AR model (Kay and Marple, 1981). The Levinson-Durbin recursive algorithm was utilized to estimate the coefficients of the AR model and the variance of the white noise. The number of coefficients *p* was chosen according to the Akaike's figure of merit in the range from 8 to 14. The power spectral density was computed from the AR coefficients and from the variance of the white noise according to the maximum entropy spectral estimation approach (Kay and Marple, 1981). The AR spectral density was factorized into spectral components, the sum of which provides the entire power spectral density (Baselli et al., 1997). The AR spectral decomposition provided power and central frequency of the components of the AR spectral density. The central frequency of the components expressed in cycles^.^beats^−1^ was converted into Hz by dividing the values by μ_HP_. A spectral component was labeled as low frequency (LF) or high frequency (HF) if its central frequency ranged between 0.04 and 0.15 Hz or was in the range of ±0.05 Hz around the paced breathing rate respectively. The LF and HF powers were computed as the sum of the powers of all LF and HF spectral components respectively. The HF power of the HP series, expressed in absolute units (i.e., ms^2^) and labeled as HFa_HP_, was utilized as a marker of vagal modulation directed to the heart (Akselrod et al., 1981), while the LF power of the SAP series, expressed in absolute units (i.e., mmHg^2^) and labeled as LFa_SAP_, was utilized as a marker of sympathetic modulation directed to vessels (Pagani et al., 1986).

### Construction of surrogates and surrogate analysis

We tested the null hypotheses of HP-SAP and HP-R coupling without or with preservation of the HP information storage. This test was performed by creating two sets of surrogates.

The first set was composed by the original SAP and R series, while the HP sequence was substituted with a series obtained by randomly shuffling the HP samples (Palus, 1997). The shuffling procedure was performed according to one of the N! permutations of the HP samples. As a consequence the SAP and R series were fully uncoupled to the HP shuffled dynamics and the original HP information storage was destroyed, while preserving the distribution of all series and the repetitive dynamical structures of SAP and R. This surrogate will be referred to as HP-shuffled surrogate.

The second set was composed by time-shifted versions of the original series (Andrzejak et al., 2003). While the HP series was left unmodified, the SAP and R sequences were shifted according to a delay much larger than the maximal order of the multivariate model (i.e., 50 cardiac beats), thus destroying the short-term temporal correspondence of the SAP and R samples to HP values, while preserving the HP information storage. The delays from SAP and R to HP were independently chosen. The values at the end of the SAP and R sequences were wrapped to their onset. This surrogate will be referred to as time-shifted surrogate.

For each triplet of original HP, SAP, and R series we created a triplet of HP-shuffled and time-shifted surrogates. If the values derived from the original data were significantly different from those obtained from the surrogate sets the null hypothesis of HP-SAP and HP-R coupling without or with preservation of the HP information storage was rejected.

Surrogate data are available through the corresponding author.

### Calculation of the information-theoretic quantities

The HP series was modeled via ARX_1_X_2_, ARX_1_, ARX_2_, X_1_X_2_, and AR models where *X*_1_ = SAP and *X*_2_ = R. The delays from SAP and R to HP, τ_SAP_ and τ_R_, were set to 0 to allow the description of the fast vagal reflex (within the current HP) capable to modify HP in response to changes of SAP and R (Eckberg, 1976; Baselli et al., 1994; Porta et al., 2012a). The coefficients were identified via a traditional least squares approach and Cholesky decomposition method (Soderstrom and Stoica, 1988; Baselli et al., 1997). The AR and X parts had the same model order *p*. The model order was optimized in the range from 4 to 16 according to the Akaike figure of merit for multivariate processes (Akaike, 1974) over the most complex model structure (i.e., the ARX_1_X_2_, model). The whiteness of the HP prediction error and its mutual uncorrelation, even at zero lag, with the SAP and R series was checked over the same model (Baselli et al., 1997; Porta et al., 2012a). All remaining model structures were separately identified using the optimal order of the ARX_1_X_2_ model. After the identification of the model coefficients the variances of the prediction errors were computed and the indexes PE_HP_, SE_HP_, JTE_SAP, R → HP_, CJTE_SAP, R → HP|R_, CJTE_SAP, R → HP|SAP_, ITE_SAP, R → HP_, CSE_HP|SAP, R_, ${\text{SE}}_{\text{HP}}^{\text{SAP},\text{R}}$, ${\text{SE}}_{\text{HP}}^{\text{SAP}}{|}_{\text{R}}$, ${\text{SE}}_{\text{HP}}^{\text{R}}{|}_{\text{SAP}}$, ${\text{ISE}}_{\text{HP}}^{\text{SAP},\text{R}}$, ITE%_SAP, R → HP_, and ${\text{SE\%}}_{\text{HP}}^{\text{SAP},\text{R}}$ were evaluated.

### Statistical analysis

We performed paired *t*-tests to check the significance of the difference between time, frequency and PE_HP_ indexes derived at REST and during HDT. If the normality test (Kolmogorov-Smirnov test) was not fulfilled, the Wilcoxon signed rank test was utilized. The same test was exploited to check the difference between original and surrogate sets. Differences among the terms of the decompositions of PE_HP_ (i.e., SE_HP_ and JTE_SAP, R → HP_), of JTE_SAP, R → HP_ (i.e., CJTE_SAP, R → HP|R_, CJTE_SAP, R → HP|SAP_, and ITE_SAP, R → HP_), of SE_HP_ (i.e., CSE_HP|SAP, R_ and ${\text{SE}}_{\text{HP}}^{\text{SAP},\text{R}}$) and of ${\text{SE}}_{\text{HP}}^{\text{SAP},\text{R}}$ (i.e., ${\text{SE}}_{\text{HP}}^{\text{SAP}}{|}_{\text{R}}$, ${\text{SE}}_{\text{HP}}^{\text{R}}{|}_{\text{SAP}}$, and ${\text{ISE}}_{\text{HP}}^{\text{SAP},\text{R}}$) were assessed within the index and experimental condition via two-way repeated measures analysis of variance (Holm-Sidak test for multiple comparisons). The same test was utilized to compare ITE%_SAP, R → HP_ and ${\text{SE\%}}_{\text{HP}}^{\text{SAP},\text{R}}$ in both experimental conditions. Statistical analysis was carried out using a commercial statistical program (Sigmaplot, ver.11.0, Systat Software, San Jose, CA, USA). A *p* < 0.05 was always considered as significant.

## Results

Table 1 summarizes the results relevant to the time and frequency domain analyses of the HP and SAP series. HDT did not affect the time domain parameters of both HP and SAP series (i.e., μ_HP_, σ${}_{\text{HP}}^{2}$, μ_SAP_, and σ${}_{\text{SAP}}^{2}$). Conversely, the frequency domain indexes were significantly modified. Indeed, HFa_HP_ increased during HDT, while LFa_SAP_ significantly declined.

**Table 1:** **Results of time and frequency domain analyses of HP and SAP series**.

	**REST**	**HDT**
μ_HP_ [ms]	937 ± 99	955 ± 111
$\sigma_{\text{HP}}^{2}$ [ms^2^]	1052 ± 742	950 ± 594
HFa_HP_ [ms^2^]	144 ± 133	205 ± 150^#^
μ_SAP_[mmHg]	128 ± 21	132 ± 21
$\sigma_{SAP}^{2}$[mmHg^2^]	24.5 ± 11.6	20 ± 14.7
LFa_SAP_ [mmHg^2^]	7.6 ± 7.7	3.4 ± 3.0^#^

μ_HP_, HP mean; $\sigma_{\text{HP}}^{\text{2}}$, HP variance; HFa_HP_, HP power in HF band expressed in absolute units; μ_SAP_, SAP mean; $\sigma_{\text{SAP}}^{\text{2}}$, SAP variance; LFa_SAP_, SAP power in LF band expressed in absolute units; REST, resting supine condition; HDT, −25° head-down tilt. Values are expressed as mean ± standard deviation. The symbol

# *indicates a significant difference with p < 0.05*.

**Figure 2:**
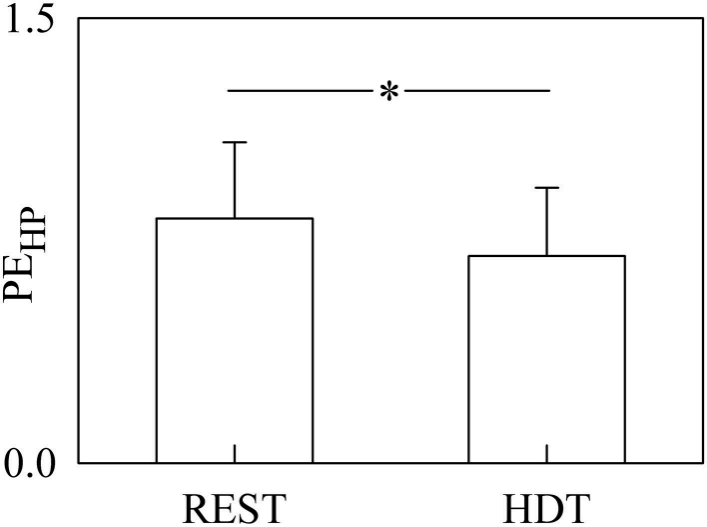
**The bargraph shows the PE_HP_ assessed at REST and during HDT**. The values are reported as mean plus standard deviation. The symbol * indicates a significant difference with *p* < 0.05.

**Figure 3:**
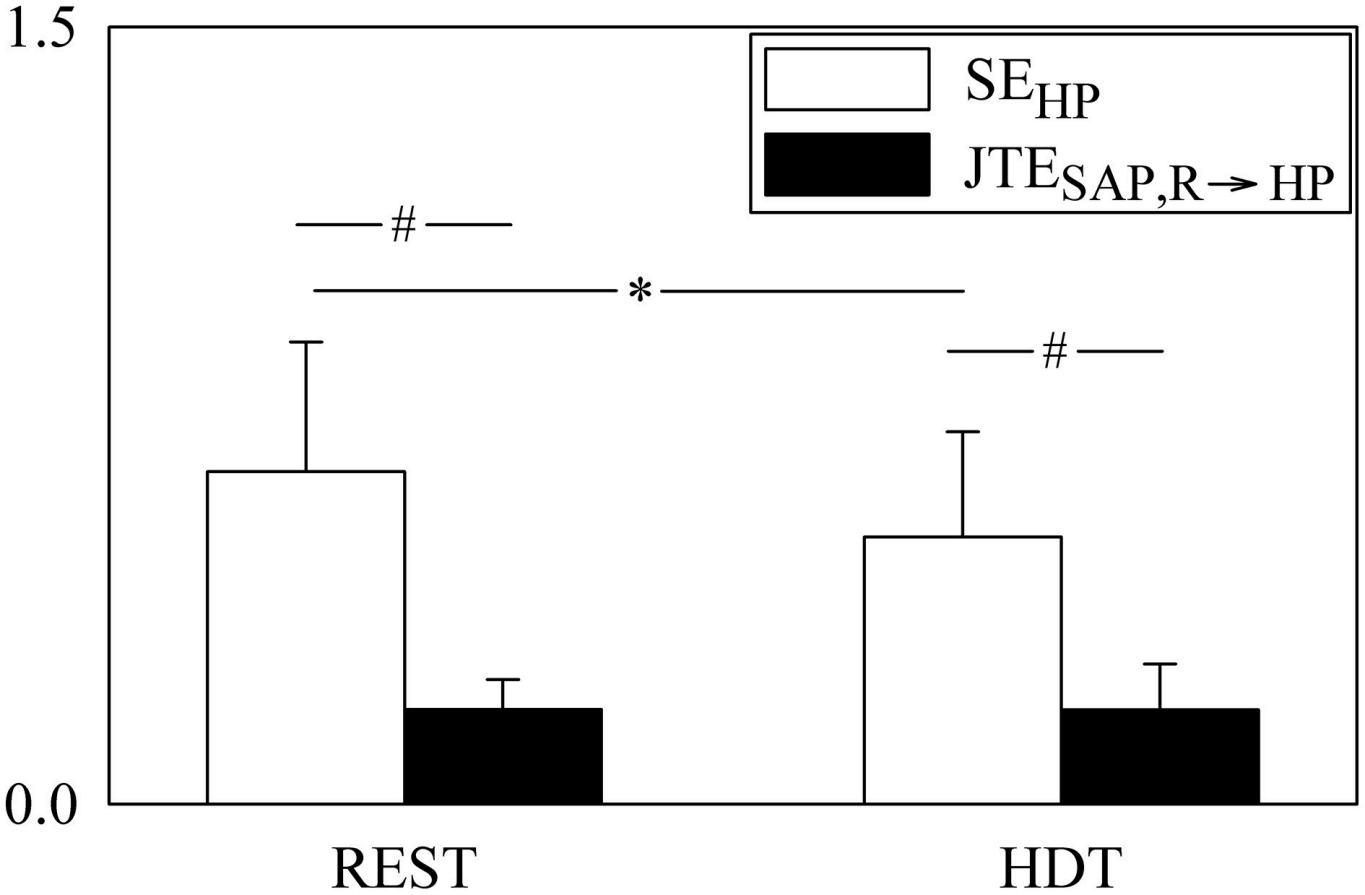
**The grouped bargraph shows the SE_HP_ (white bars) and JTE_SAP, R → HP_ (black bars) assessed at REST and during HDT**. SE_HP_ and JTE_SAP, R → HP_ are the two terms of the PE_HP_ decomposition according to Equation (20). The values are reported as mean plus standard deviation. The symbol * indicates a significant difference between experimental conditions within the same index, while the symbol # indicates a significant difference between indexes within the same experimental condition with *p* < 0.05.

**Figure 4:**
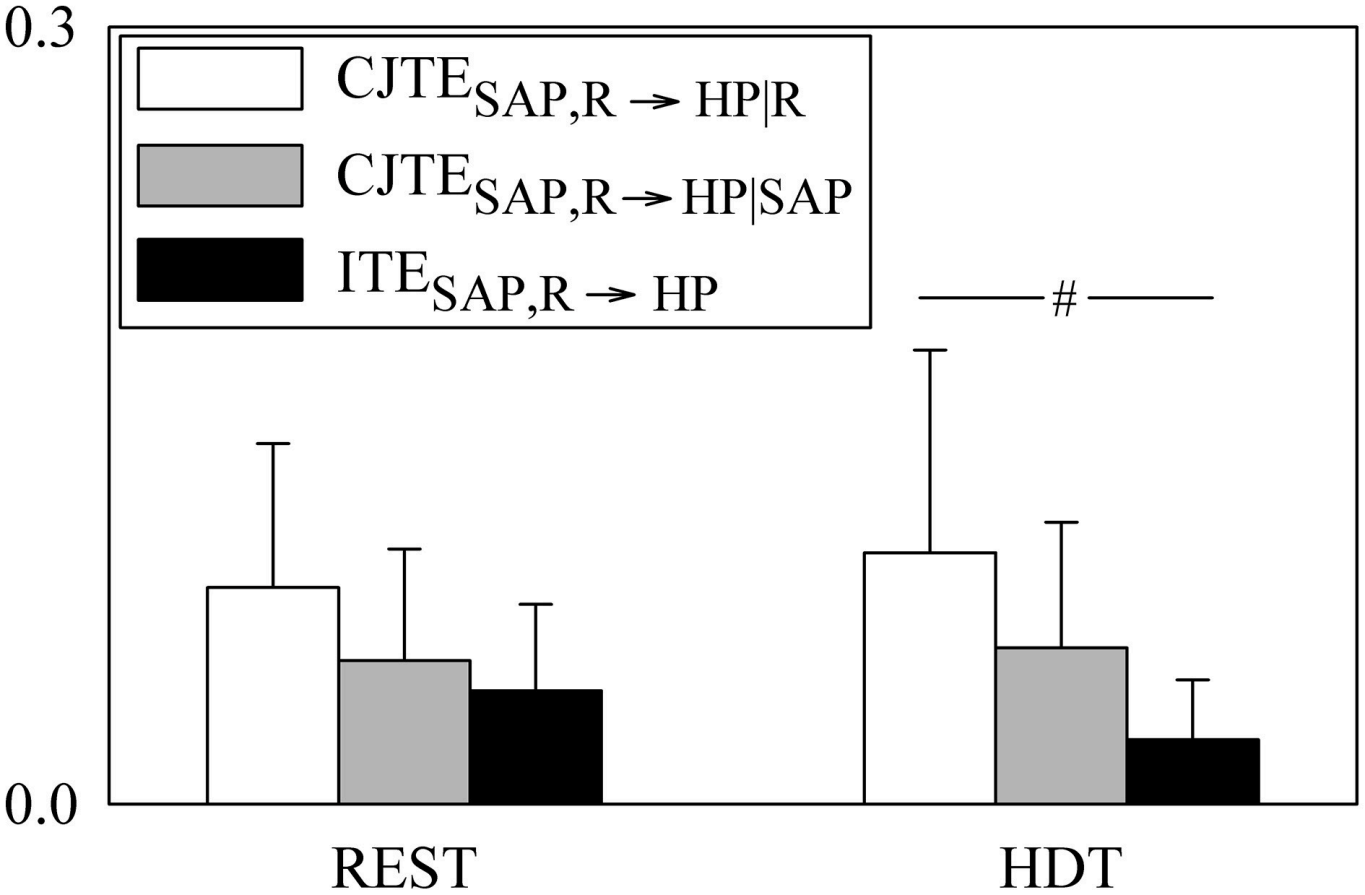
**The grouped bargraph shows the CJTE_SAP_, R → HP|R (white bars), CJTE_R, SAP → HP|SAP_ (gray bars) and ITE_SAP, R → HP_ (black bars) assessed at REST and during HDT**. CJTE_SAP, *R* → HP|R_, CJTE_R, *SAP* → HP|SAP_, and ITE_SAP, R → HP_ are the three terms of the JTE_SAP, R → HP_ decomposition according to Equation (21). ITE_SAP, R → HP_ is the synergistic/redundant term of the JTE_SAP, R → HP_ decomposition. The values are reported as mean plus standard deviation. The symbol # indicates a significant difference between indexes within the same experimental condition with *p* < 0.05.

**Figure 5:**
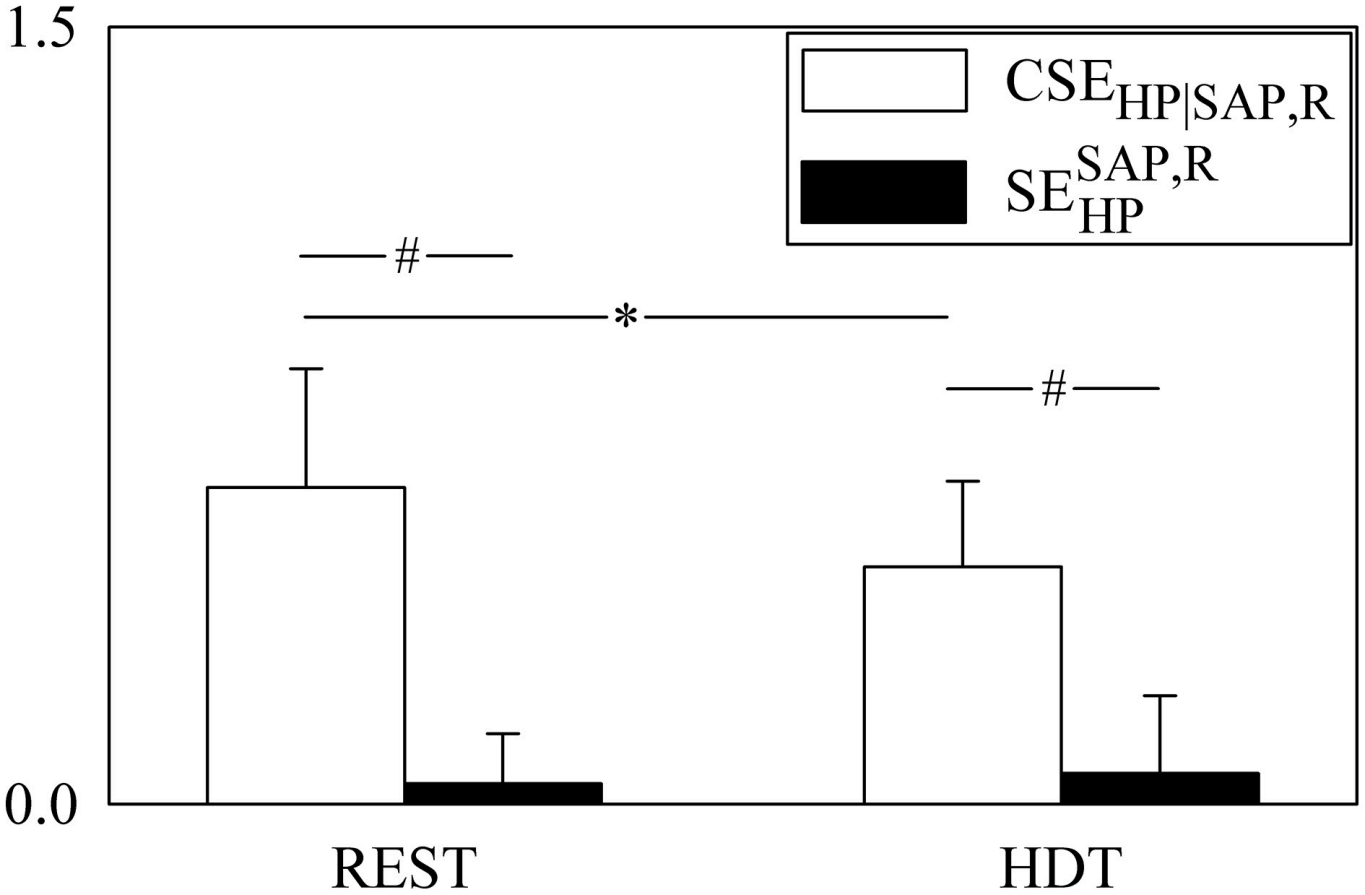
**The grouped bargraph shows the CSE_HP|SAP, R_ (white bars) and ${\text{SE}}_{\text{HP}}^{\text{SAP,R}}$ (black bars) assessed at REST and during HDT**. CSE_HP|SAP, R_ and ${\text{SE}}_{\text{HP}}^{\text{SAP},\text{R}}$ are the two terms of the SE_HP_ decomposition according to Equation (13). ${\text{SE}}_{\text{HP}}^{\text{SAP},\text{R}}$ is the synergistic/redundant term of the SE_HP_ decomposition. The values are reported as mean plus standard deviation. The symbol * indicates a significant difference between experimental conditions within the same index, while the symbol # indicates a significant difference between indexes within the same experimental condition with *p* < 0.05.

**Figure 6:**
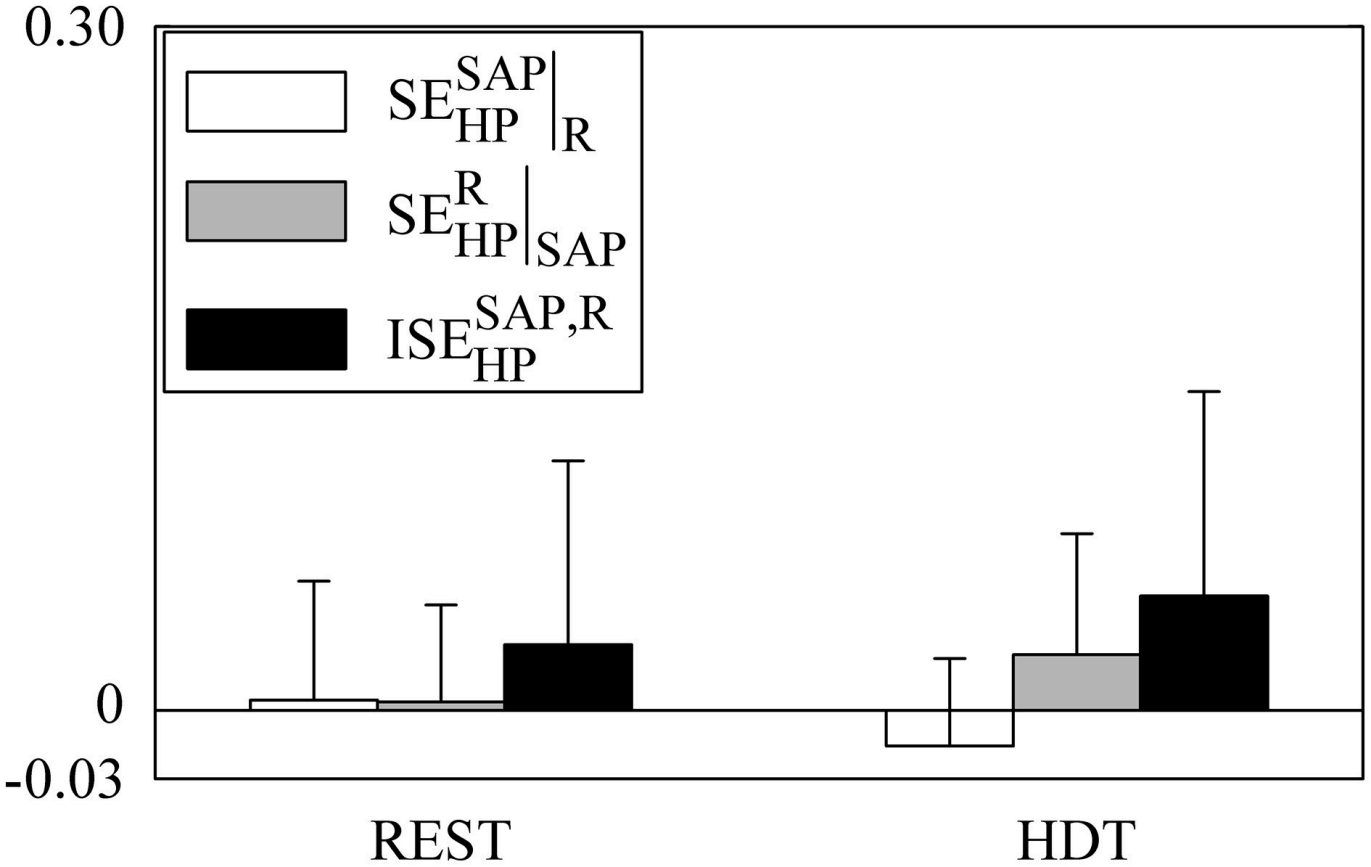
**The grouped bargraph shows the ${\text{SE}}_{\text{HP}}^{\text{SAP}}{\text{|}}_{\text{R}}$ (white bars), ${\text{SE}}_{\text{HP}}^{\text{R}}{\text{|}}_{\text{ SAP}}$ (gray bars), and ${\text{ISE}}_{\text{HP}}^{\text{SAP,R}}$ (black bars) assessed at REST and during HDT**. ${\text{SE}}_{\text{HP}}^{\text{SAP}}{|}_{\text{R}}$, ${\text{SE}}_{\text{HP}}^{\text{R}}{|}_{\text{SAP}}$, and ${\text{ISE}}_{\text{HP}}^{\text{SAP},\text{R}}$ are the three synergistic/redundant terms of the SE_HP_ decomposition according to Equation (22). The values are reported as mean plus standard deviation.

The bar graph shown in Figure 2 compares PE_HP_ computed at REST and during HDT. PE_HP_ was significantly decreased during HDT. The full decomposition of PE_HP_ into more specific quantities is given in Figures 3–**6**.

The grouped bar graph of Figure 3 shows the two terms forming PE_HP_ according to Equation (20) (i.e., SE_HP_, white bars, and JTE_SAP, R → HP_, black bars). SE_HP_ was markedly larger than JTE_SAP, R → HP_ in both conditions. SE_HP_ significantly decreased during HDT, while JTE_SAP, R → HP_ was not affected by the posture modification.

The grouped bar graph of Figure 4 depicts the three constituents of JTE_SAP, R → HP_ according to Equation (21) (i.e., CJTE_SAP, R → HP|R_, white bars, CJTE_SAP, R → HP|SAP_, gray bars, and ITE_SAP, R → HP_, black bars). None of the terms forming JTE_SAP, R → HP_ were modified by HDT. At REST CJTE_SAP, R → HP|R_, CJTE_SAP, R → HP|SAP_, and ITE_SAP, R → HP_ were similar, while during HDT ITE_SAP, R → HP_ was significantly smaller than CJTE_SAP, R → HP|R_. It is remarkable that ITE_SAP, R → HP_ was larger than 0 in all subjects regardless of the experimental condition, suggesting that SAP and R contributed redundantly to JTE_SAP, R → HP_ both at REST and during HDT.

The grouped bar graph of Figure 5 shows the two terms forming SE_HP_ according to Equation (13) (i.e., CSE_HP|SAP, R_, white bars, and ${\text{SE}}_{\text{HP}}^{\text{SAP},\text{R}}$, black bars). CSE_HP|SAP, R_ significantly decreased during HDT, while ${\text{SE}}_{\text{HP}}^{\text{SAP},\text{R}}$ was not affected by the posture modification. ${\text{SE}}_{\text{HP}}^{\text{SAP},\text{R}}$ was significantly smaller than CSE_HP|SAP, R_ in both experimental conditions. The average value of ${\text{SE}}_{\text{HP}}^{\text{SAP},\text{R}}$ was larger than 0 indicating that, on average, SAP and R contributed redundantly to the information storage in HP. However, ${\text{SE}}_{\text{HP}}^{\text{SAP},\text{R}}$ was negative in 31% and 46% of subjects at REST and during HDT respectively, thus indicating that at the level of the information storage in HP synergy between SAP and R occurred frequently.

The grouped bar graph of Figure 6 depicts the synergistic/redundant terms present in the decomposition of SE_HP_ according to Equation (22) (i.e., ${\text{SE}}_{\text{HP}}^{\text{SAP}}{|}_{\text{R}}$, white bars, ${\text{SE}}_{\text{HP}}^{\text{R}}{|}_{\text{SAP}}$, gray bars, and ${\text{ISE}}_{\text{HP}}^{\text{SAP},\text{R}}$, black bars). No significant difference was detected within indexes given REST or HDT or within experimental conditions given the index. On average, ${\text{SE}}_{\text{HP}}^{\text{SAP}}{|}_{\text{R}}$, ${\text{SE}}_{\text{HP}}^{\text{R}}{|}_{\text{SAP}}$, and ${\text{ISE}}_{\text{HP}}^{\text{SAP},\text{R}}$ were positive at REST, but indexes were negative in 54%, 31%, and 54% of subjects respectively. On average during HDT ${\text{SE}}_{\text{HP}}^{\text{R}}{|}_{\text{SAP}}$ and ${\text{ISE}}_{\text{HP}}^{\text{SAP},\text{R}}$ remained positive, while ${\text{SE}}_{\text{HP}}^{\text{SAP}}{|}_{\text{R}}$ became negative. ${\text{SE}}_{\text{HP}}^{\text{SAP}}{|}_{\text{R}}$, ${\text{SE}}_{\text{HP}}^{\text{R}}{|}_{\text{SAP}}$, and ${\text{ISE}}_{\text{HP}}^{\text{SAP},\text{R}}$ were negative in 77%, 38%, and 38% of subjects respectively during HDT. These results stressed that at the level of the information storage in HP synergy between SAP and R was commonly present both at REST and during HDT, even though it did not take priority over redundancy.

**Figure 7:**
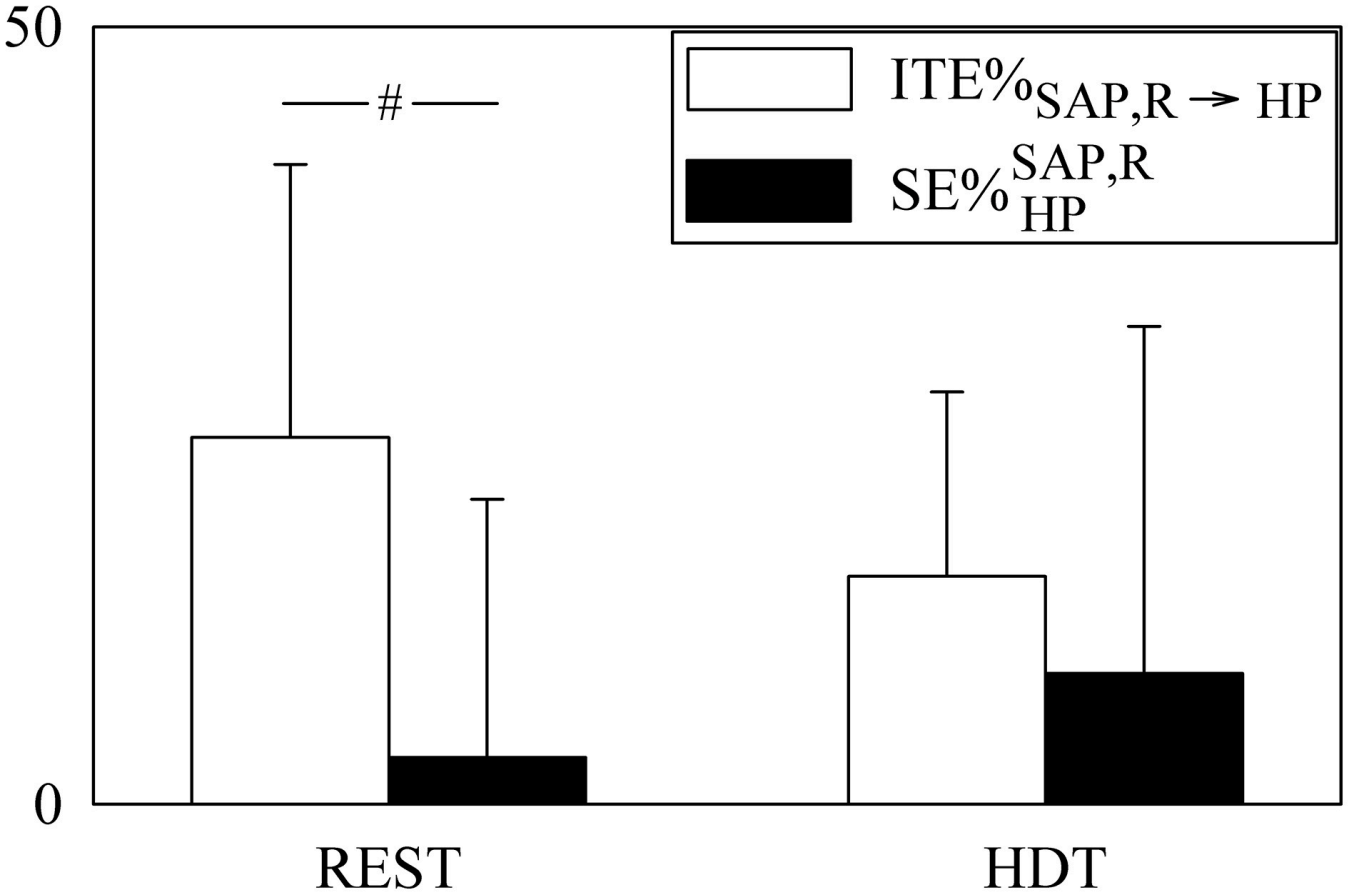
**The grouped bargraph shows the ITE%_SAP, R_→HP (white bars) and ${\text{SE\%}}_{\text{HP}}^{\text{SAP,R}}$ (black bars) assessed at REST and during HDT**. ITE%_SAP, R → HP_ and ${\text{SE\%}}_{\text{HP}}^{\text{SAP},\text{R}}$ measure the percent redundant/synergistic contributions of SAP and R to JTE_SAP, R → HP_ and SE_HP_ respectively. The values are reported as mean plus standard deviation. The symbol # indicates a significant difference between indexes within the same experimental condition with *p* < 0.05.

The contributions of the redundant/synergistic terms to JTE_SAP, R → HP_ and SE_HP_ (i.e., ITE_SAP, R → HP_ and ${\text{SE}}_{\text{HP}}^{\text{SAP},\text{R}}$) are compared in Figure 7 after expressing them as ITE%_SAP, R → HP_ and ${\text{SE\%}}_{\text{HP}}^{\text{SAP},\text{R}}$. Since ITE%_SAP, R → HP_ was larger than 20% at REST, ITE_SAP, R → HP_ represented a sizable amount of JTE_SAP, R → HP_. Conversely, ${\text{SE\%}}_{\text{HP}}^{\text{SAP},\text{R}}$ was significantly smaller (about 3%), being a negligible quantity compared to SE_HP_. Both ITE%_SAP, R → HP_ and ${\text{SE\%}}_{\text{HP}}^{\text{SAP},\text{R}}$ were not affected by HDT. Remarkably, while ITE%_SAP, R → HP_ was consistently positive in all subjects, the variability of ${\text{SE\%}}_{\text{HP}}^{\text{SAP},\text{R}}$ was much higher due to the presence of both negative and positive values.

For the surrogate analysis, the PE_HP_ was significantly larger when computed over the original series than over both types of surrogates. This result held in the case of any term of the JTE_SAP, R → HP_ decomposition according to Equation (21). The SE_HP_ and, more specifically, CSE_HP|SAP, R_ was significantly larger over the original data than HP-shuffled surrogates. Conversely, the terms of the ${\text{SE}}_{\text{HP}}^{\text{SAP},\text{R}}$ decomposition according to Equation (23) computed over the original data were similar to those calculated over both types of surrogates.

## Discussion

The methodological findings of this study can be summarized as follows: (i) the study proposes a full decomposition of PE of *y* in Ω = {*y*,*x*_1_,*x*_2_} that includes the decomposition of SE of *y* in addition to the known decomposition of JTE from *x*_1_ and *x*_2_ to *y*; (ii) both JTE and SE decompositions include a term describing redundancy/synergy of *x*_1_ and *x*_2_ in contributing to the information carried by *y* and the redundant/synergistic term of SE has a more complex structure; (iii) the utility of the JTE and SE decompositions is demonstrated in the field of cardiovascular control analysis to disentangle physiological mechanisms from spontaneous variations and clarify the origin of the increase of respiratory sinus arrhythmia during HDT.

The experimental findings of this study can be summarized as follows: (i) in agreement with the literature we confirm the increase of respiratory sinus arrhythmia after acute cardiopulmonary loading induced by HDT; (ii) the PE of HP decreases during HDT, thus suggesting a larger complexity of the cardiac control and a vagal enhancement; (iii) the SE of HP is larger than the JTE from SAP and R to HP and the SE of HP due to SAP and R is negligible compared to the CSE of HP given SAP and R both at REST and during HDT, thus emphasizing the relevance of physiological mechanisms other than those mediated by SAP and R changes in governing the HP dynamics; (iv) the invariance of the CJTE from SAP and R to HP given R and CJTE from SAP and R to HP given SAP during HDT suggests a limited involvement of the baroreflex and cardiopulmonary pathway in controlling the HP dynamic during HDT; (v) the decrease of the CSE of HP given SAP and R in response to HDT suggests that the increase of respiratory sinus arrhythmia might be the consequence of modifications of the sinus node dynamical properties and/or an enhanced activity of the respiratory centers occurring independently of the cardiac baroreflex and cardiopulmonary circuits; (vi) SAP and R contribute redundantly to the information jointly transferred to HP, while, at the level of the information storage into HP, redundancy and synergy are more balanced; (vii) the amount of synergy or redundancy of SAP and HP to the information transfer and storage in HP is not affected by HDT, thus again stressing that the response to HDT is not mediated by a strong involvement of the cardiac baroreflex and cardiopulmonary circuits; (viii) surrogate analysis indicates that the terms of the JTE decomposition detect significant HP-SAP and HP-R interactions and the CSE of HP given SAP and R measures a significant HP information storage both at REST and during HDT.

### Methodological findings

#### Decomposition of PE of y in Ω = {y,x_1_,x_2_}

The study proposes a viable approach to tackle the issue of the PE decomposition of a target signal *y* affected by two exogenous signals, *x*_1_ and *x*_2_. The PE, measuring the reduction of uncertainty about the present of *y* when past samples of *y, x*_1_, and *x*_2_ are given (Chicharro and Ledberg, 2012; Faes et al., 2015), can be decomposed into the information jointly transferred from *x*_1_ and *x*_2_ to *y* and the information stored into *y*. The information stored into *y* estimates the amount of uncertainty about the present of *y* that can be resolved using only past values of *y* (Lizier et al., 2012; Wibral et al., 2014), while the JTE from *x*_1_ and *x*_2_ to *y* quantifies the reduction of uncertainty about the present value of *y* when past samples of *x*_1_ and *x*_2_ are given above and beyond the information stored into *y* (Porta et al., 2015). The decomposition of JTE from *x*_1_ and *x*_2_ to *y* into two terms considering the contribution of *x*_1_ to *y* given *x*_2_ and that of *x*_2_ to *y* given *x*_1_plus an additional term describing the balance between redundancy and synergy to the joint information transfer (i.e., the ITE) was originally proposed in (Stramaglia et al., 2012) by generalizing to the conditional case the notion of interaction information (McGill, 1954). Conversely, the full decomposition of SE of *y* is original, thus completing the PE decomposition of *y* in Ω *=* {*y*,*x*_1_,*x*_2_}. The SE is first decomposed into two terms: (i) the first term is the SE of *y* conditioned on the exogenous sources (i.e., the CSE of *y* given *x*_1_ and *x*_2_) assessing the information storage not attributable to SAP and R influences; (ii) the second term is a synergistic/redundant term indicating whether past values of *y* and of both exogenous signals, when jointly considered, contribute synergistically or redundantly to resolve the uncertainty of *y* (i.e., the SE of *y* due to *x*_1_ and *x*_2_). The SE of *y* due to *x*_1_ and *x*_2_ can be decomposed further into two terms considering the unique contribution of *x*_1_ and *x*_2_ plus an extra term describing the joint contribution of *x*_1_ and *x*_2_ (i.e., the ISE). We stress that, while in the decomposition of JTE from *x*_1_ and *x*_2_ to *y* given in Equation (21) the synergistic/redundant term is only one (i.e., ITE), in the decomposition of SE of *y* given in Equation (22) only one term is definitely larger than or equal to zero (i.e., the CSE of *y* given *x*_1_ and *x*_2_). Indeed, the SE of *y* due to *x*_1_ and *x*_2_ can be positive or negative and, consequently, the SE of y larger or smaller than the CSE of *y* given *x*_1_ and *x*_2_, depending on the balance among synergistic/redundant behaviors of past values of *y* and *x*_1_, past values of *y* and *x*_2_ and past values of *y* and both *x*_1_ and *x*_2_ in reducing the uncertainty of *y*.

#### Both JTE and SE of y in Ω = {y,x_1_,x_2_} feature redundant/synergistic terms

The JTE decomposition includes a term describing the redundant or synergistic contribution of *x*_1_ and *x*_2_ to JTE (McGill, 1954; Stramaglia et al., 2012; Barrett, 2015; Wibral et al., 2015). This term indicates that the single contributions of *x*_1_ and *x*_2_ to JTE do not invariably contain duplicate information (i.e., redundant contribution) about the present value of *y*, but, conversely, the contemporaneous knowledge of *x*_1_ and *x*_2_ might lead to extra information (i.e., synergistic contribution). The inclusion of any new additional exogenous signal in the universe of knowledge (e.g., *x*_2_ in Ω\*x*_2_) leading to synergy means to improve the prediction of *y* well above the one obtained when the exogenous signals are taken individually. Through, the SE decomposition this study proves that the notion of synergy or redundancy applies to the information storage as well. Information storage depends on the action of exogenous inputs (Lizier et al., 2012; Wibral et al., 2014) and the significance of the contribution of the exogenous signals to SE of *y* has been proved experimentally in the context of cardiovascular control analysis (Porta et al., 2015). The SE decomposition indicates that *x*_1_ and *x*_2_ might contribute redundantly or synergistically to the information storage. The study of the synergistic/redundant contributions of *x*_1_ and *x*_2_ to the information stored in *y* is more complex because the assessment of synergy/redundancy should take into account not only the ability of past values of *x*_1_ and *x*_2_ to reduce the uncertainty of *y* but also that of past values of *y*, thus increasing the number of synergistic/redundant terms. Conversely, in the case of the information transfer synergy/redundancy is exclusively attributable to past values of *x*_1_ and *x*_2_ since the contribution of past values of *y* is conditioned out. The multiplicity of terms describing redundancy/synergy at the level of the information storage has been spelled out in Equation (23) and viable estimators for their computation were provided.

#### JTE and SE decompositions in network physiology

Our approach is framed into the emerging field of network physiology describing the complexity of aggregates of parts and their interactions as a network of nodes with interconnections (Bashan et al., 2012). This feature is in common with other approaches adopting the same logic for representing complex interactions among subsystems regardless of the scale (David et al., 2006; Bressler and Seth, 2011; Bashan et al., 2012; Iatsenko et al., 2013; Kralemann et al., 2014; Stankovski et al., 2015; Porta and Faes, 2016). This description might involve the utilization of raw data (David et al., 2006; Bressler and Seth, 2011), realizations of point processes or series of events (Porta and Faes, 2016) or phase evolutions estimated from raw data or series of events (Iatsenko et al., 2013; Kralemann et al., 2014; Stankovski et al., 2015). The functionals exploited to assess the strength of the interconnections among nodes might be fully adherent to the Wiener-Granger principle (Granger, 1963) if their calculation is based on a direct comparison of indexes computed in the unrestricted and restricted universes of knowledge via metrics assessing the predictability improvement (Bressler and Seth, 2011; Porta and Faes, 2016) and/or uncertainty decrement (Schreiber, 2000; Hlavackova-Schindler et al., 2007; Porta and Faes, 2016), or based on the explicit computation of coupling functions (Iatsenko et al., 2013; Kralemann et al., 2014; Stankovski et al., 2015), or the estimation of coupling coefficients of an assigned model (David et al., 2006). The approach devised in this study is fully consistent with the Wiener-Granger principle in the information domain, where functionals assess the uncertainty decrement and account for conditioning variables according to a multivariate approach. As such, some analogs can be found with fully multivariate methods based on the explicit calculation of coupling functions (Kralemann et al., 2014; Stankovski et al., 2015) and on its decomposition into self-, direct, and indirect components (Stankovski et al., 2015). Nevertheless, in the present study the proposed decomposition is achieved in a completely different framework (i.e., the Wiener-Granger one) and it is expressively devised for the identification of synergistic/redundant components, rather than for the exclusive separation of the direct influences from the indirect ones.

### Experimental findings

#### Time and frequency domain analyses of HP dynamics during HDT

Time and frequency domain parameters confirmed that HDT does not significantly affect the HP and SAP means (Harrison et al., 1986; Nagaya et al., 1995; Kardos et al., 1997; Tanaka et al., 1999) but it increases the HF power of HP (Kardos et al., 1997) and decreases the LF power of SAP (Weise et al., 1995). These findings were interpreted as a sign of the involvement of the autonomic nervous system in adjusting HP and SAP in response to the posture challenge and, more specifically, as an indication of the increased vagal modulation directed to the sinus node and the decreased sympathetic modulation directed to the vessels during HDT (Nagaya et al., 1995; Weise et al., 1995; Tanaka et al., 1999). Unfortunately, time and frequency domain analyses exploited in this study, and traditionally utilized to understand the physiological adaptation to acute central circulatory hypervolaemia (Weise et al., 1995; Kardos et al., 1997), are not helpful to clarify the origin of the increase of respiratory sinus arrhythmia during HDT. This limitation can be primarily attributable to the univariate nature of these classical time and frequency domain analyses and to their inability to interpret causality, thus preventing the possibility of disentangling the HP response to HDT driven by changes of SAP and R from the one independent of them. This limitation is tackled by the proposed multivariate approach grounded in the framework of information dynamics.

#### Information dynamics approach to the assessment of the cardiovascular control during HDT

The involvement of the cardiovascular control in regulating the HP dynamic during HDT is clearly suggested by the significant decrease of PE of HP. Since we reported earlier that the level of predictability of HP based on past samples of HP, SAP and R is under vagal control being increased during head-up tilt and high dose administration of atropine (Porta et al., 2012c), the decrease of the amount of uncertainty about the present of HP that can be resolved by past values of HP, SAP, and R, as measured by the PE of HP, suggests a larger complexity of the cardiac control and an increased vagal regulation during HDT. Even though based on multivariate analysis, this finding is useless in explaining the mechanisms underpinning vagal activation and the increase of respiratory sinus arrhythmia because PE is a global parameter vaguely linked to physiological mechanisms. We need to directly exploit the decomposition of JTE of SAP and R to HP and SE of HP to try to elucidate the origin of the increase of respiratory sinus arrhythmia during HDT.

The information stored into HP, as measured by the SE of HP, is significantly larger than the JTE of SAP and R to HP. This finding suggests that, even though the knowledge of SAP and R is really helpful in resolving the uncertainty of HP, the contribution of these two signals is significantly smaller compared to the ability of past HP values in predicting the current HP. The relevance of the information storage in HP as the likely consequence of the importance of signals driving HP dynamics independently of SAP and R, such as modulation of efferent cardiac neural activity driven by central commands, originating from respiratory and vasomotor centers in the brainstem, and independent of afferent inputs (Preiss and Polosa, 1974; Valentinuzzi and Geddes, 1974; Preiss et al., 1975; Koepchen, 1984; Dick et al., 2009). Also the dynamical properties of the sinus node (i.e., how it responds to changes of sympathetic and vagal inputs) might play an important role in setting the magnitude of the information stored in HP because they directly affect the memory of HP over its past values (Chess and Calaresu, 1971; Berger et al., 1989; Porta et al., 2003). In addition to the dynamical response of receptors, the self-storage of information into HP depends on the type of neurotransmitters, their concentration, rate of release, degradation and removal and sympatho-vagal interactions (Kawada et al., 1996; Nakahara et al., 1998, 1999; Porta et al., 2003).

Remarkably, we found that the SE of HP and, more specifically, the CSE of HP given SAP and R decreased during HDT, while the JTE of SAP and R to HP remained unmodified. The reduction of the CSE of HP given SAP and R supports the *central drive* hypothesis as a possible explanation for the increase of respiratory sinus arrhythmia during HDT. Indeed, the activation of a central mechanism, independent of the cardiac baroreflex and cardiopulmonary stimulation, could limit the ability of past values of HP in reducing the uncertainty of the current HP value. For example, if the respiratory centers improved their activity (Valentinuzzi and Geddes, 1974; Dick et al., 2009), the resulting augmented modulation of the cardiovagal motorneuron responsiveness would produce an increase of respiratory sinus arrhythmia (Eckberg, 2003) and make cardiac regulation more complex (Porta et al., 2012c).

The invariance of JTE from SAP and R to HP during HDT is not a sufficient condition to exclude the role of the cardiac baroreflex and cardiopulmonary reflexes in the rise of respiratory sinus arrhythmia during HDT. Indeed, during graded head-up tilt we found that the invariance of JTE from SAP and R to HP hides the progressive increase of the information transferred from SAP to HP in Ω, as quantified by the CJTE from SAP and R to HP given R, and the decrease of the information transferred from R to HP in Ω, as quantified by the CJTE from SAP and R to HP given SAP, with the magnitude of the orthostatic challenge and baroreflex unloading (Porta et al., 2015). Therefore, it is necessary to check the trend of CJTE from SAP and R to HP given R and CJTE from SAP and R to HP given SAP during HDT to better characterize the involvement of the baroreflex and cardiopulmonary pathway in controlling HP dynamics. Given the invariance of the CJTE from SAP and R to HP given R and CJTE from SAP and R to HP given SAP during HDT we conclude that the amount of information transferred along the cardiac baroreflex and cardiopulmonary reflexes is not significantly different from that observed at REST and, thus, we exclude again the cardiac baroreflex control and cardiopulmonary reflexes as possible physiological mechanisms underpinning the observed increase of respiratory sinus arrhythmia.

The opposite influences on the venous return, central blood volume and central venous pressure during head-up tilt and HDT, leading to baroreflex unloading, sympathetic activation, and vagal withdrawal in the case of the head-up tilt (Montano et al., 1994; Cooke et al., 1999; Furlan et al., 2000; Marchi et al., 2013) and cardiopulmonary loading and sympathetic inhibition in the case of HDT (Nagaya et al., 1995; Tanaka et al., 1999), might suggest opposite effects on the degree of involvement of the baroreflex control of HP and cardiopulmonary neural circuits. Contrary to this expectation, while head-up tilt maneuver led to an augmented involvement of the baroreflex control of HP and a reduced participation of the cardiopulmonary reflexes (Porta et al., 2012b, 2015), the invariance of the information transferred along the cardiac baroreflex and cardiopulmonary pathway observed in the present study suggests that the physiological response to acute central circulatory hypervolaemia during HDT cannot be simply deduced from the knowledge of the response to acute central circulatory hypovolemia during head-up tilt.

#### Redundancy and synergy of SAP and R in contributing to the information transferred and stored in HP dynamics

SAP and R contribute redundantly to the information jointly transferred to HP. This means that SAP and R hold common information about the present value of HP above and beyond that derived from past values of HP. Remarkably, this quantity is important since it explains more than 20% of JTE from SAP and R to HP. The redundant nature of the SAP and R contributions to the information transferred to HP is not surprising. Indeed, R can directly modulate SAP by modifying venous return, pressure gradients over large arteries in the thorax and stroke volume via respiratory-related changes of the intrathoracic pressure (Innes et al., 1993; Toska and Eriksen, 1993; Caiani et al., 2000). However, the redundant contribution of SAP and R to JTE from SAP and R to HP might also come from more complex interactions and integrations between vasomotor and respiratory centers occurring at the brain stem level. This amount of redundancy might accomplish a principle of fault tolerance and harmonization of neural responses. In this specific experimental protocol the amount of redundancy of SAP and R to the information transferred to HP was not significantly varied during HDT, again confirming that HDT did not affect quantities closely linked to the functioning of the cardiac baroreflex and cardiopulmonary reflexes.

Even though on average SAP and R contribute redundantly to the information storage into HP, we cannot conclude that redundancy is prevailing over synergy as far as the information storage of HP is concerned. Indeed, the SE of HP due to SAP and R, measuring the balance between redundancy and synergy at the level of the information storage at REST, is quite small (i.e., 3% of the SE of HP) and in 31% of subjects synergistic behaviors between SAP and R in contributing to the SE of HP were observed. As a result of the presence of both redundancy and synergy inside the group of subjects in both experimental conditions, indexes describing the synergistic/redundant behavior of SAP and R to the information storage of HP are characterized by greater variability compared to the index describing the synergistic/redundant behavior of SAP and R to the information transferred into HP. The amount of redundancy of SAP and R to the information storage of HP was not significantly varied during HDT, thus again stressing that HDT did not affect quantities linked to the functioning of the cardiac baroreflex and cardiopulmonary circuits even when the action of these reflexes is mediated by memory effects of HP on its own past.

#### Surrogate analysis

Surrogate data were constructed with the main aim to test the significance of the proposed indexes as markers of the strength of the HP-SAP and HP-R coupling in absence or presence of a significant amount of information stored in HP. According to this idea two types of surrogates, both destroying the HP-SAP and HP-R coupling are generated. The first type, the HP-shuffled surrogates, wiped out the HP autocorrelation function, while the second type, the time-shifted surrogates, preserved it. Remarkably, all indexes derived from the decomposition of JTE from SAP and R to HP both at REST and during HDT were significantly larger from those derived from surrogates, regardless of the type. This result indicates that both at REST and during HDT the HP-SAP and HP-R interactions are significant as well as the detected redundancy of SAP and R in contributing to the JTE from SAP and R to HP, suggesting that indexes derived from the JTE decomposition are helpful to detect physiological interactions from spontaneous variations. The SE of HP and, more specifically, the CSE of HP given SAP and R, was significantly larger in the original data than in the HP-shuffled surrogates both at REST and during HDT. This result suggests that the information stored into the HP dynamics is significant in both experimental conditions. Conversely, the terms of the decomposition of SE of HP due to SAP and R computed over the original data were indistinguishable from those calculated over surrogate data regardless the type of surrogate both at REST and during HDT. We suggest two possible explanations for this finding: (i) the contributions of SAP and R to the SE of HP did not reach the level of significance both at REST and during HDT; (ii) the surrogate analysis proposed in the present study is not suitable to test the significance of the causal interactions from SAP and R to HP at the level of the SE decomposition.

#### Significance of the study and future perspectives

A full decomposition of the amount of uncertainty about a target signal that can be resolved based on two presumed driving signals is provided. The decomposition is relevant to the information jointly transferred from the two driving signals to the target one and to the information stored into the destination signal. Terms describing the balance between redundancy and synergy of the two driving series in resolving the uncertainty of the target signal have been highlighted and viable estimators have been proposed. The application to the experimental data suggests the relevance of the approach in dissecting out cardiovascular control mechanisms with the aim of accepting or rejecting physiological hypotheses. Since the proposed quantities are highly specific and take the form of indexes that can be computed very efficiently and robustly via a traditional multivariate regression analysis of spontaneously varying variables, they appear to be suitable candidates for large scale applications to clinical databases recorded even under uncontrolled conditions. Due to the generality of the approach it might be applied not only to cardiovascular physiology and neuroscience, but also in any field of science in which interactions among systems, or constituents of the same system, are under evaluation. Future studies should extend the decomposition to model-free frameworks to account for the possible presence of nonlinear dynamics disregarded by the present approach. In addition, given that in the present contribution the interaction terms actually represent the balance between redundancy and synergy, future studies might test different information decomposition strategies (Barrett, 2015; Wibral et al., 2015) and extend the proposed decomposition of SE to allow the coexistence of both redundancy and synergy as independent positive quantities. We also advocate studies devoted to the improvement of the physical/physiological interpretation of the parts of the JTE and SE decompositions that can be achieved by extending the application of these decompositions to new experimental conditions, proposing new experiments aimed at modulating the terms of the decompositions, comparing this approach to different techniques for the quantification of the coupling strength, developing new strategies for the construction of ad-hoc surrogate sets and designing specific simulation studies.

### Conflict of interest statement

The authors declare that the research was conducted in the absence of any commercial or financial relationships that could be construed as a potential conflict of interest.
